# Burden of illness in patients with chronic hypoparathyroidism not adequately controlled with conventional therapy: a Belgium and the Netherlands survey

**DOI:** 10.1007/s40618-020-01442-y

**Published:** 2020-10-30

**Authors:** N. A. T. Hamdy, B. Decallonne, P. Evenepoel, D. Gruson, L. van Vlokhoven-Verhaegh

**Affiliations:** 1 Department of Medicine, Division of Endocrinology, and Centre for Bone Quality, Leiden University Medical Center, Albinusdreef 2, 2333 ZA Leiden, The Netherlands; 2 Department of Endocrinology, University Hospitals Leuven and KU Leuven, Leuven, Belgium; 3 Department of Microbiology, Immunology and Transplantation, University Hospitals Leuven and KU Leuven, Leuven, Belgium; 4 Department of Clinical Biochemistry, Cliniques Universitaires Saint-Luc and Université Catholique de Louvain, Brussels, Belgium; 5 Department of Medical Affairs, Shire Netherlands BV, a Takeda company, Amsterdam, The Netherlands

**Keywords:** Chronic hypoparathyroidism, Clinical manifestations, Comorbidities, Conventional therapy, Inadequate control, Physician survey

## Abstract

**Purpose:**

To determine the burden of illness in patients with not adequately controlled chronic hypoparathyroidism receiving conventional therapy in Belgium and the Netherlands.

**Methods:**

Data were generated from a cross-sectional, two-part online survey where endocrinologists from both countries and nephrologists from Belgium were invited by phone to participate. Part 1 included collecting data on general management of patients with hypoparathyroidism. In Part 2, physicians were requested to provide data on one or two current cases of patients with chronic hypoparathyroidism not adequately controlled on conventional therapy. Data collected included aetiology of hypoparathyroidism, clinical manifestations, comorbidities, results of laboratory and other investigations used for diagnosis and screening for complications, therapy received, and physician’s perception of impaired quality of life (QoL).

**Results:**

Thirty-six endocrinologists and 29 nephrologists from Belgium and 28 endocrinologists from the Netherlands participated in the survey. Data included clinical symptoms, biochemical parameters, and QoL for 97 current patients with not adequately controlled chronic hypoparathyroidism on conventional therapy. Median duration of not adequately controlled hypoparathyroidism was 2.2 years, range 0.17–20.0. Most patients had neuromuscular (85%) and/or neurological (67%) symptoms, 71% had abnormal biochemical parameters, 10% were overweight, and physicians perceived that 71% had impaired QoL. Most frequently reported comorbidities included hypertension (25%), renal comorbidity (20%), diabetes mellitus (12%), and dyslipidaemia (11%).

**Conclusion:**

Patients with chronic hypoparathyroidism not adequately controlled on conventional therapy experience a substantial burden of illness, mainly due to persistence of symptoms and presence of multiple comorbidities.

**Electronic supplementary material:**

The online version of this article (10.1007/s40618-020-01442-y) contains supplementary material, which is available to authorized users.

## Introduction

Chronic hypoparathyroidism is a rare endocrine disorder caused by the absence or insufficient production of parathyroid hormone (PTH), the major regulator of calcium homeostasis [1, 2], leading to the classical biochemical features of hypocalcaemia and hyperphosphataemia [1–3]. In the kidney, PTH stimulates reabsorption of calcium, promotes phosphate excretion, and enhances the conversion of 25-hydroxyvitamin D to its active metabolite 1,25-dihydroxyvitamin D, which increases transport of calcium and phosphate in intestinal cells [4]. PTH also stimulates bone resorption, which mobilises calcium and phosphorous into the circulation [4]. Hypocalcaemia is responsible for most of the neuromuscular symptoms and signs associated with hypoparathyroidism, while hyperphosphataemia and an elevated calcium-phosphate product contribute to ectopic soft tissue calcifications [1–3, 5].

The most common cause of hypoparathyroidism is iatrogenic, following neck surgery in about 75% of cases [5–7]. Other less common causes of hypoparathyroidism include idiopathic, congenital, or autoimmune disease [1, 3, 5]. Functional hypoparathyroidism may be due to hypomagnesaemia, which inhibits PTH secretion, or be due to end-organ hyporesponsiveness as seen in chronic kidney disease [5, 8]. The prevalence of chronic hypoparathyroidism is not well defined. It was variably estimated to be 37/100,000 individuals in the United States, 10.2/100,000 in Norway, 5.3/100,000 in Italy, and 2.3/100,000 in Denmark [3, 9–11].

Conventional treatment of hypoparathyroidism consists of oral calcium supplements and active vitamin D preparations, which corrects serum calcium levels and controls symptoms of hypoparathyroidism in many patients [12, 13], but fails to provide adequate or consistent control of biochemical parameters in a number of them. Even when adequate biochemical control is achieved, conventional treatment may not fully or even partially reverse some of the symptoms of chronic hypoparathyroidism leading to persistent morbidity and impaired quality of life (QoL) [1, 5, 13].

Patients with hypoparathyroidism are at risk of short-term and long-term complications and comorbidities, such as hypocalcaemia-related neuromuscular, cardiovascular, or cognitive manifestations [1, 2, 13–15]. Conventional therapy may also be associated with side effects, such as hypercalcaemia, hypercalciuria, nephrocalcinosis, nephrolithiasis, and decreased renal function [13, 15].

Patients with hypoparathyroidism are less likely to be employed full- or part-time and are more likely to have reduced work productivity, and to have symptoms preventing them from carrying out normal daily activities, all of which may negatively affect their relationships [16]. A recent study conducted in 13 countries showed an inverse relationship between patient self-rated overall symptom severity and QoL and health status, as assessed using SF-36 and EQ-5D-5L questionnaires, and increased caregiver burden [17]. Taken together, the physical, cognitive, and emotional symptoms and morbidities associated with not adequately controlled hypoparathyroidism thus represent a substantial burden of illness that adversely affects QoL [16–20].

The main objective of this study was to evaluate the yet to be ascertained burden of illness in patients with not adequately controlled chronic hypoparathyroidism on conventional therapy, as surveyed among a group of hospital-based treating physicians from Belgium and the Netherlands.

## Methods

### Part 1: Physician survey on general management of chronic hypoparathyroidism

An online physician survey was developed by three endocrinologists (N.A.T. Hamdy, B. Decallonne, and R. Peeters) and a nephrologist (P. Evenepoel), all with expertise in the management of patients with hypoparathyroidism, and a specialist in laboratory medicine (D. Gruson), in collaboration with representatives of Shire, a Takeda company, and STETHOS International, a Healthcare Market Research company. All registered endocrinologists in Belgium and the Netherlands and registered nephrologists in Belgium were initially contacted by telephone to determine their interest in participating in the survey, in which case, they would be required to be either personally treating patients with chronic hypoparathyroidism or supervising colleagues treating these patients and to be involved in treatment decisions concerning management of their hypoparathyroidism. Physicians who expressed an interest received a written invitation to complete the questionnaire (detailed document in Supplementary Materials), and the offer of a modest financial incentive for participating by the industry study sponsor Shire, part of Takeda, in accordance with industry standards.

In this survey, ‘chronic hypoparathyroidism’ was defined as hypocalcaemia in the presence of inappropriately normal or low PTH, as confirmed by repeated laboratory measurements, requiring treatment with active vitamin D, and to be of genetic, autoimmune, or idiopathic aetiology, or persisting for > 12 months after neck surgery. For the purpose of this survey, ‘not adequately controlled hypoparathyroidism’ was defined as the persistence of clinical manifestations of hypocalcaemia, and/or of one or more of the following biochemical manifestations: hypocalcaemia, significant fluctuations in serum calcium, hyperphosphataemia, or hypercalciuria, despite perceived optimal compliance with conventional treatment.

Data collected from the survey included the specialty of treating physicians (endocrinology or nephrology), country of practice (Belgium or the Netherlands), type of institution (academic or non-academic hospital), number of patients with chronic hypoparathyroidism treated in the department of the responding physician, number of patients with not adequately controlled hypoparathyroidism on conventional treatment, and any additional tests performed for diagnosis or screening for complications (Survey Sect. 1, Supplementary Material).

### Part 2: Physician survey on patients with not adequately controlled chronic hypoparathyroidism

Participating physicians were asked to provide clinical and biochemical data on one or two unique cases of real-life patients they were currently treating for chronic hypoparathyroidism who were not adequately controlled on conventional therapy (Survey Sect. 3, Supplementary Material). No other instructions were provided to the physicians on which patients to select for reporting. To better represent the hypoparathyroidism patient population, patient cases were weight-adjusted according to the number of not adequately controlled patients followed by the responding physician at the time of completing the survey.

### Data collection and reporting

*Clinical features* Data on cause of hypoparathyroidism and main causes for inability to control the disease, clinical manifestations, abnormal biochemical parameters and other screening parameters for diagnosis and identification of potential complications were collected for all patients at their last visit to the treating physician.

*Quality of life data* Data collected on QoL were based on treating physician-perceived patient impairment in QoL at their last visit.

*Pharmacological treatment* Data on the dose of calcium supplement, dose and type of active vitamin D (alfacalcidol or calcitriol), and use of thiazide diuretics to control hypercalciuria were also collected from the last visit.

*Statistical testing* No formal statistical analysis was performed, and data are reported as mean ± SD unless otherwise stated.

## Results

### Part 1: General survey on the management of chronic hypoparathyroidism

Of the 93 physicians participating in the survey, the majority (29 nephrologists and 36 endocrinologists) were from Belgium, with just under one-third (28 endocrinologists) from the Netherlands (Table 1). Of the 65 respondents from Belgium, 13 were from Brussels, 24 from Flanders, and 28 from Wallonia. Most respondents (71%) were affiliated to non-academic hospitals, 56% of whom were endocrinologists (Table 1). Besides blood sampling, the most frequently ordered test in the follow-up of patients with chronic hypoparathyroidism was the measurement of 24-h urinary calcium excretion (performed in 77% of cases). Thiazide diuretics were prescribed to 8% of patients.

**Table 1:** Profile of responding physicians and aetiology of chronic hypoparathyroidism by country

Parameter	Belgium	Netherlands	Total
Responding physicians, *n*	65	28	93
Type of hospital, *n* (%)			
Academic	20 (31)	7 (25)	27 (29)
Non-academic	45 (69)	21 (75)	66 (71)
Hospital department of responding physician, *n* (%)			
Endocrinology	34 (52)	18 (64)	52 (56)
Nephrology	28 (43)	0	28 (30)
General internal medicine	3 (5)	9 (32)	12 (13)
Other	0	1 (4)	1 (1)
Aetiology of hypoparathyroidism, %			
Neck surgery	79.4	76.0	77.7
Idiopathic	7.9	6.8	7.4
Congenital	5.2	6.7	5.9
Autoimmune disease	3.5	6.9	5.1
Irradiation	1.0	3.5	2.2
Not known	3.0	0.3	1.7
Patients with chronic HypoPT treated in hospital department, mean, *n*			
Endocrinologist	20.9^a^	47.2^b^	–
Nephrologist	10.9^c^	–	–
Patients with chronic HypoPT not adequately controlled in hospital department, mean, *n*			
Endocrinologist	4.5^a^	7.5^b^	–
Nephrologist	3.9^c^	–	–
Patients ‘not adequately controlled’ per hospital department, %			
Endocrinologist	21.5^a^	15.9^b^	–
Nephrologist	35.8^c^	–	–

*HypoPT *hypoparathyroidism

^a^Endocrinologists, *n* = 36

^b^Endocrinologists, *n* = 28

^c^Nephrologists, *n* = 29

### Part 2: Physician-reported demographic and clinical characteristics of patients with not adequately controlled chronic hypoparathyroidism

Respondent physicians provided data for 97 patients with chronic hypoparathyroidism not adequately controlled on conventional therapy: 60 cases were provided by physicians from Belgium and 37 by those from the Netherlands. Demographic and clinical characteristics of these patients are reported in Table 2. Most patients were women, with an average age of 49 years, many of whom had long-lasting not adequately controlled hypoparathyroidism at the time of the survey. Median duration of chronic hypoparathyroidism from time of diagnosis was 5.1 years, range 0.33–47.9, and reported median duration of not adequately controlled hypoparathyroidism was 2.2 years, range 0.17–20.0, after the stipulated year after diagnosis of hypoparathyroidism. The most common cause of hypoparathyroidism was neck surgery (67% of patients), performed a mean of 9.5 ± 10.4 years before the survey.

**Table 2:** Demographic and clinical characteristics of patients with not adequately controlled hypoparathyroidism

Parameter	Total sample (*N* = 97)
Sex, %	
Male	34
Female	66
Age, years	
Mean (SD)	48.5 (16.8)
Range, %	
≤ 30	20
31–40	17
41–50	16
51–60	22
61–70	15
≥ 71	10
Aetiology of hypoparathyroidism, % of patients	
Neck surgery	67
Autoimmune	11
Idiopathic	10
Congenital	7
Irradiation	2
Unknown	3
Years since diagnosis, % of patients	
≤ 2	30
> 2–5	19
> 5–10	17
> 10–20	22
> 20	12
Median (range), years	5.1 (0.33–47.9)
Duration in years of not adequately controlled hypoparathyroidism^a^, % of patients	
< 2	39
2–5	32
5–10	15
10–20	15
> 20	0
Median (range), years	2.2 (0.17–20.0)
Treatment and dose at time of diagnosis, mean (SD)^b^	
Calcium,^c^ mg/day	2300 (1352)
Alfacalcidol,^d^ µg/day	2 (2.2)
Calcitriol,^e^ µg/day	2 (2.2)

*SD *standard deviation

^a^Persisting hypoparathyroidism > 12 months after diagnosis

^b^*n* = 66

^c^*n* = 64

^d^*n* = 36

^e^*n* = 26

According to treating physicians’ opinion, the most frequently reported reasons for not adequately controlled hypoparathyroidism were poor compliance (41%), limited treatment choice (25%), comorbidities (21%), and side effects of treatment for hypoparathyroidism (21%). Sixty-nine patients (71%) had comorbidities at diagnosis, not necessarily specific for hypoparathyroidism, including hypertension (25%), renal comorbidity (20%), diabetes mellitus (12%), dyslipidaemia (11%) and overweight (10%). Patients with renal comorbidity were exclusively followed by nephrologists in Belgium and many had concomitant morbidities, such as hypertension (76%), dyslipidaemia (43%), diabetes mellitus (39%), and overweight (26%).

In response to the survey question ‘*What were the treatments prescribed after the diagnosis of chronic hypoparathyroidism was established?*’ physicians involved in establishing the diagnosis of chronic hypoparathyroidism or who had access to the patient’s clinical records from the time of diagnosis (*n* = 66) reported that the percentage of patients who received treatment at the time of diagnosis was 97% for calcium supplements, 94% for active vitamin D (55% alfacalcidol and 39% calcitriol), and 7% for thiazide diuretics.

**Fig. 1:**
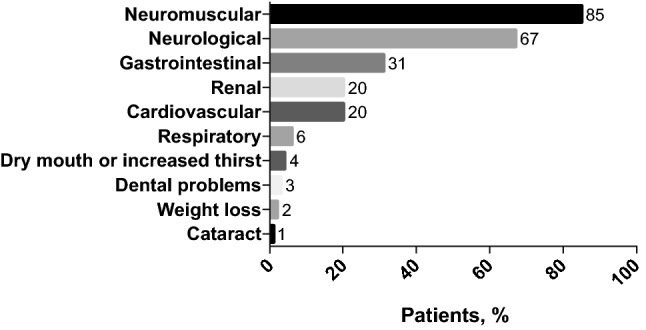
Clinical manifestations reported in real-life patients with not adequately controlled chronic hypoparathyroidism currently receiving conventional therapy (*N* = 97). Results are expressed as the percentage of patients with the clinical manifestation. *Signs and symptoms are listed in Table 3

In response to the survey question ‘*What clinical manifestations (symptoms/signs) of chronic hypoparathyroidism does this patient currently have?*’ the most commonly reported symptoms among the 97 patients were neuromuscular (85%) and neurological (67%) (Fig. 1), although there was likely overlap between these two categories of symptoms. The frequency of individual manifestations within broader grouping terms is shown in Table 3.

**Table 3:** Frequency of clinical manifestations of hypoparathyroidism in patients with not adequately controlled chronic hypoparathyroidism.^a^

Grouping term	Manifestation	Total sample, % (*N* = 97)
Neuromuscular	Cramps	67
	Paraesthesia	62
	Tetany attacks	9
	Chvostek’s sign	8
	Trousseau’s sign	7
	Seizures	4
	Cerebral calcifications	4
	Laryngospasm	3
	Bronchospasm	3
	Severe respiratory disorders	1
Cardiovascular	Palpitations	15
	Arrhythmia	3
	Heart failure	3
Gastroenterological	Constipation	21
	Abdominal cramps	12
Neurological	Fatigue	45
	Anxiety	24
	Mood swings	22
	Concentration disorders	18
	Depression	11
	Sleep disorders	7
	Confusion	4
	Dementia	1
Renal	Renal failure	16
	Renal calcifications (nephrocalcinosis)	7
	Renal stones	4
	Polyuria	1
Respiratory	Shortness of breath	4
	Throat tightness	2
	Wheezing	1
Others	Dry mouth or increased thirst	4
	Fracture	4
	Dental problems	3
	Weight loss	2
	Cataract	1
	Infection	0
	Papilloedema	0
	Other, please specify	4

^a^Signs and symptoms listed were from a predefined list included in the questionnaire

Patients generally remained on the treatment regimen prescribed at diagnosis, although doses of both active vitamin D and calcium were required to be increased in 47% of patients, calcium alone in 14% of patients, and active vitamin D alone in 13% of patients. At the time of the survey, 96% of patients were receiving calcium supplementation and 97% active vitamin D: 51% alfacalcidol and 46% calcitriol. In Belgium, alfacalcidol, calcitriol, and ergocalciferol/cholecalciferol were respectively prescribed by endocrinologists to 27%, 72%, and 11% of patients (*n* = 38), and by nephrologists to 33%, 47%, and 20% of patients (*n* = 22). Treatments to control hypoparathyroidism are shown by country in Supplementary Table 1.

In response to the survey question *‘How often do you monitor biochemical parameters in this patient?*’ physicians reported that patients had laboratory parameters evaluated on average five times a year. The frequency of evaluation was every month in 16%, every two months in 17%, every three months in 40%, twice a year in 19%, once a year in 6%, and other in 3%.

**Fig. 2:**
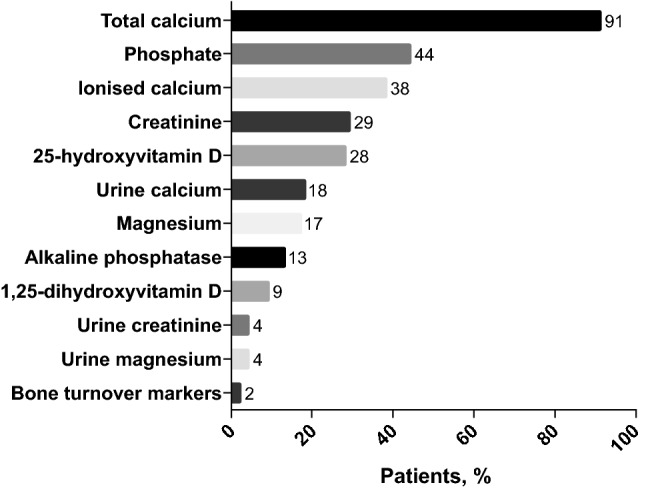
Biochemical parameters above or below the normal laboratory reference range in patients with not adequately controlled chronic hypoparathyroidism on conventional therapy (*n *= 68). Results are expressed as the percentage of patients with the parameter outside the normal range

To determine the percentage of patients with chronic hypoparathyroidism on conventional therapy with abnormal biochemical parameters, response to the survey question ‘*Does this patient currently still have biochemical parameters outside the normal laboratory reference ranges?’* revealed that 71% of patients (*n* = 68) had laboratory values above or below the normal reference range at the time of the survey. The most frequently reported laboratory values outside the normal range are shown in Fig. 2, and are detailed by country in Supplementary Table 2.

**Fig. 3:**
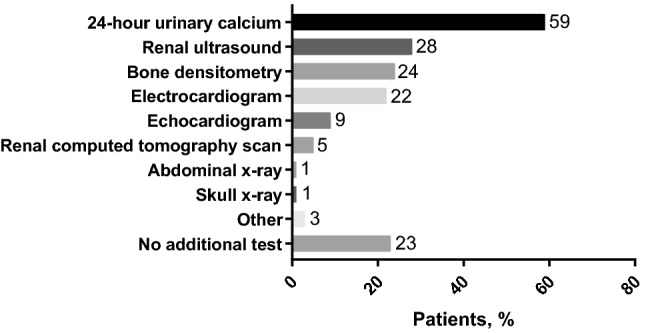
Responses to the survey question ‘*Which additional tests do you currently carry out to monitor hypoparathyroidism?*’ (*N* = 97). Results are expressed as the percentage of patients who had the test

Responses to the survey question ‘*Which additional tests do you currently carry out to monitor the hypoparathyroidism?’* are shown in Fig. 3. No additional tests were reported to be performed to monitor hypoparathyroidism in 23% of patients with not adequately controlled disease. In Belgium, a higher percentage of patients had an additional test when treated by a nephrologist compared with an endocrinologist (bone densitometry, 50% vs 32%; electrocardiogram, 37% vs 29%; echocardiogram, 32% vs 5%; renal computed tomography scan, 25% vs 0%) because of the inherent increased risk of other morbidities associated with renal comorbidity. Conversely, a 24-h urinary calcium measurement was requested by 66% of endocrinologists compared with 45% of nephrologists.

Over the 12 months before the survey, the inability to control chronic hypoparathyroidism was the primary reason for hospitalisation in 17% of patients. Among patients who were hospitalised (*n* = 16), mean number of hospitalisations was 2.1 ± 1.5 (median, 2.0) over the 12 months before the survey, and mean duration of hospitalisation was 12.5 ± 17.0 days (median, 5.0 days). Responding physicians perceived that among patients with chronic hypoparathyroidism, 71% experienced a decrease in QoL, 12% had no change in QoL, and 17% had an improvement in QoL since the onset of hypoparathyroidism.

**Fig. 4:**
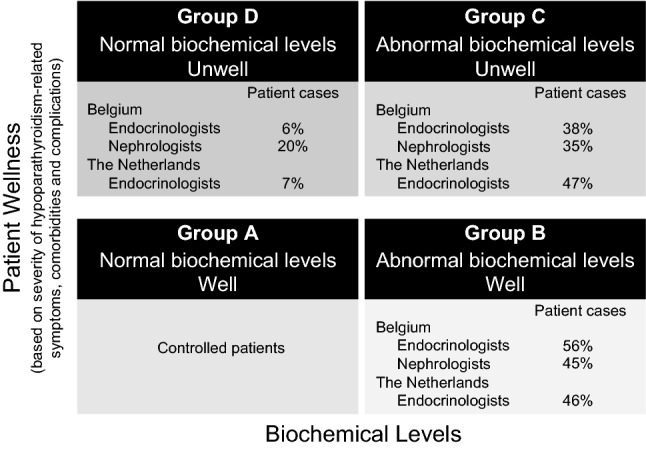
Physician profiling of symptoms and biochemical parameters of reported patients with chronic hypoparathyroidism not adequately controlled on conventional therapy. Patients (*N* = 97) were profiled as belonging to 1 of 4 predefined categories using a quadrant matrix in response to the survey question ‘*In your opinion, which profile of not adequately controlled chronic hypoparathyroidism illustrated in the following figure corresponds best with that of your patient?*’ Results are expressed as the percentage of patients assigned to a category by specialists in each country. From Belgium, endocrinologists provided 38 patient cases, and nephrologists 22 patient cases; from the Netherlands, endocrinologists provided 37 patient cases. *Physician assignments of patients to each category of not adequately controlled are no surrogate for appropriately collected epidemiological data

Physicians participating in the survey were also requested to assess the profile of their patients with not adequately controlled chronic hypoparathyroidism in terms of abnormal biochemical parameters and presence and severity of symptoms (Fig. 4) using a visual four-quadrant matrix similar to that developed to define patients with hypoparathyroidism not adequately controlled on conventional therapy [21]. Keeping in consideration that this was not a true epidemiological study, and that patients were assigned to different categories, responses showed that about 50% of patients were assigned a profile of abnormal biochemical parameters with absent or mild clinical symptoms/comorbidities.

## Discussion

This online survey, conducted among a sample of endocrinologists and nephrologists actively involved in the management of patients with chronic hypoparathyroidism of any aetiology in Belgium and the Netherlands, captured data from patients with relatively long-standing hypoparathyroidism, including the clinical features of those in whom the disease was not adequately controlled by conventional therapy. The 2015 European Society of Endocrinology (ESE) clinical guideline for the treatment of chronic hypoparathyroidism in adults states, “The aim of treatment of chronic hypoparathyroidism is to relieve symptoms of hypocalcamia and improve the patient’s quality of life,” and that “the treatment should aim to maintain serum calcium levels in the low normal range,…with patients being free of symptoms or signs of hypocalcaemia, while avoiding hypercalciuria” [22]. The development of hypercalciuria represents a significant limitation to the optimization of conventional therapy, as this usually necessitates a reduction in the dose of active vitamin D and/or calcium supplements that can often lead to undertreatment and poorly controlled hypoparathyroidism. Relevant causes of not adequately controlled hypoparathyroidism, as also reflected by analysis of data from our survey, include non-compliance and undertreatment due to concerns about side-effects or complications of treatment such as hypercalcaemia and/or hypercalciuria and associated risk of renal morbidity [5, 23]. Symptoms of hypoparathyroidism may also persist in the absence of biochemical abnormalities under treatment with conventional therapy perceived to be optimal [21].

Improving quality of life is an important treatment aim and our survey showed that patients with chronic hypoparathyroidism not adequately controlled on conventional therapy experience a substantial burden of illness particularly due to chronic symptoms, comorbidities and/or side-effects of treatment [1, 13, 15]. The most frequently reported symptoms were neuromuscular (including cramps and paraesthesia) and neurological (including fatigue and anxiety). A recent study from Belgium also reported paraesthesia and/or cramps in a substantial proportion of patients [24].

Biochemical parameters were reported to be outside the normal laboratory reference range in most patients at the most recent outpatient visit prior to the survey, including total and ionised calcium, 24-h urinary calcium excretion and serum phosphate, highlighting the difficulties in complying with the above outlined ESE Guideline for the treatment of chronic hypoparathyroidism in adults [22]. It is important to avoid therapy-induced elevated levels of calcium and phosphate in patients with chronic hypoparathyroidism because a long-standing high calcium-phosphate product (serum calcium × phosphate concentration) increases the risk of extra-skeletal calcifications, including nephrocalcinosis, nephrolithiasis, and basal ganglia calcifications [13, 22].

In line with published literature, the most commonly reported cause for hypoparathyroidism in our survey was neck surgery [4, 13]. At diagnosis, about 50% of patients with not adequately controlled hypoparathyroidism had comorbidities associated with long-standing disease or disease-related disturbances in mineral metabolism, with 17% of patients requiring a mean of 2.1 hospitalisations over the 12 months before the survey. Meola et al. reported that in patients with chronic post-operative hypoparathyroidism, conventional therapy with oral calcium and active vitamin D was not only suboptimal, but also associated with increased risk of renal complications [25]. In our survey 20% of patients had renal comorbidity, which is also in keeping with data from a recent single-centre retrospective study conducted in Belgian in-patients with not adequately controlled chronic hypoparathyroidism [24], which provided further evidence for the association of renal morbidity with poor control of hypoparathyroidism, and for the need for frequent monitoring of these patients to correct or preserve renal function and to address associated comorbidities.

In patients treated with conventional therapy, symptoms of hypoparathyroidism may persist and affect QoL despite serum calcium levels being maintained on target at the lower limit or just below the normal reference range. In this survey, 71% of treating physicians reported to have subjectively perceived deterioration of QoL in their patients since disease onset, although no validated instruments were used to evaluate QoL.

The main strengths of our survey are that selection bias was avoided by extending participation to all registered endocrinologists and nephrologists in two countries, and that it captured a sample of 93 respondent physicians involved in the care of patients with hypoparathyroidism — approximately two-thirds from Belgium and one-third from the Netherlands — who represented 85% of those who expressed an interest in taking part in the survey. However, the relatively small combined number of patients with hypoparathyroidism reported by physicians from both countries, the relative magnitude and severity of symptoms, which possibly dictated referral of these patients to these specialists, and the heterogeneity in some aspects of their care, may represent a potential limitation to the generalisability of our findings to the chronic hypoparathyroidism population at large.

The patient presentation matrix shown in Fig. 4 [21] is a tool developed to help physicians define patients with not adequately controlled chronic hypoparathyroidism. Estimates of patient numbers in each category of not adequately controlled hypoparathyroidism reflect the distribution of abnormalities in real-life patients reported by respondents to our survey. These figures should not, however, be used as surrogates for true epidemiological data about these categories of inadequate control in the population at large of patients with chronic hypoparathyroidism.

Physician-reported patient compliance to treatment was relatively low despite perceived compliance being a parameter defining not adequately controlled patients. Patients may inform their physicians they are being compliant but may not be taking their medications as prescribed. While patients not adequately controlled were defined in the survey using a range of parameters, physician opinion of a patient as not adequately controlled is based on a broader overall assessment of this complex disease that likely reflects real-world clinical experience. Comorbidities reported by physicians may lack specificity as possibly reflecting demographics of the patient population rather than being necessarily related to hypoparathyroidism. Further potential limitations are that the survey may not have fully captured certain aspects of the disease, such as real compliance to conventional treatment, or accurate assessment of QoL of patients, which were based on perceptions of the treating physicians rather than obtained using validated instruments, and that the survey design did not include a control group.

On the other hand, results from our survey identified potential opportunities for improving specific aspects of care, including consideration of patients' compliance as potential reason for poor control, which hold significant implications in the long-term welfare of patients with chronic hypoparathyroidism. Of particular relevance is the opportunity to preempt potential complications associated with the long-term use of active vitamin D preparations and calcium supplements. Only 59% of treating physicians regularly monitored 24-h urinary calcium excretion, which is mandatory to identify persistent hypercalciuria and associated risks for nephrocalcinosis, nephrolithiasis and impaired renal function [1, 23]. Our findings also reveal the generally limited monitoring of serum magnesium and 25-hydroxyvitamin D levels in follow-up.

Our findings suggest that there are some regional differences in the management of patients with chronic hypoparathyroidism between Belgium and the Netherlands (Supplementary Tables 1 and 2). These differences may be related to limited treatment choices and/or side effects of treatment, which may influence prescribing patterns. Our findings may thus not be extrapolated to other countries or other physicians treating patients with chronic hypoparathyroidism. Results from our survey outline the difficulties in fulfilling the recommendations set in the ESE clinical guideline for the general goals of management of chronic hypoparathyroidism in adults [22], even among treating physicians experienced in the management of this disorder. Better adherence to these guidelines will hopefully pave the way for better control of hypoparathyroidism, with achievement of treatment targets, while appropriately monitoring for complications and side-effects of treatment, thus minimising the burden of illness and improving quality of life.

In conclusion, results from this physician survey show that patients with chronic hypoparathyroidism not adequately controlled on conventional therapy experience a substantial burden of illness, mainly due to the persistence of symptoms of hypoparathyroidism, comorbidities, and the need for closely monitoring these conditions. We believe our findings hold significant clinical implications for the management of patients with chronic hypoparathyroidism by being instrumental in increasing awareness of the burden of illness associated with failure to adequately control the disease and of the need for optimising treatment to alleviate or prevent this burden.

## Electronic supplementary material

Below is the link to the electronic supplementary material.

Supplementary file1 (PDF 14 kb)

Supplementary file1 (PDF 1115 kb)

## Data Availability

All aggregated datasets (but not individual participants’ data) generated, analysed, and used to support the results of the survey reported in this article (text, tables, figures, and appendices) are available for sharing upon reasonable request to the corresponding author.
