# Supplementary material for: Burden of illness in patients with chronic hypoparathyroidism not adequately controlled with conventional therapy: a Belgium and the Netherlands survey
Source: J Endocrinol Invest. 2020 Oct 30;44(7):1437–46. doi: 10.1007/s40618-020-01442-y (PMC8195792; doi:10.1007/s40618-020-01442-y)
Supplement: Supplementary file 1 — Supplementary file1 (PDF 14 kb) [file 40618_2020_1442_MOESM1_ESM.pdf]

Supplementary Table 1. Country-specific differences in prescribing patterns for patients with not adequately controlled chronic hypoparathyroidism at the time of the survey

	**Belgium n=60**	**Netherlands n=37**
Patient, %		
Calcium supplement	96	96
Alfacalcidol	29	81
Calcitriol	64	23
Ergocalciferol/Cholecalciferol	14	23

Supplementary Table 2. Country-specific differences in abnormal biochemical parameters in patients with not adequately controlled chronic hypoparathyroidism

	**Belgium**<br>**n=46**	**Netherlands**<br>**n=21**
Patients, %		
Total calcium	93	89
Phosphate	61	16
Ionised calcium	36	40
Creatinine	35	17
25-hydroxy vitamin D	39	10
Urine calcium	26	5
Magnesium	26	2
Alkaline phosphatase	17	7
1,25-hydroxy vitamin D	14	0
Urine creatinine	7	0
Urine magnesium	7	0
Bone turnover markers	4	0
