# Supplementary material for: Burden of illness in patients with chronic hypoparathyroidism not adequately controlled with conventional therapy: a Belgium and the Netherlands survey
Source: J Endocrinol Invest. 2020 Oct 30;44(7):1437–46. doi: 10.1007/s40618-020-01442-y (PMC8195792; doi:10.1007/s40618-020-01442-y)
Supplement: Supplementary file 2 — Supplementary file1 (PDF 1115 kb) [file 40618_2020_1442_MOESM2_ESM.pdf]

SCREENER

S1/ What is your specialty?

- ❑ (Internist-) Nephrologist
- ❑ (Internist-) Endocrinologist
- ❑ *General Internist*
- ❑ *General/Endocrine surgeon*
- ❑ Other ➔ **EXCLUDE**

Programming: 1 answer only.

S2/ Details of the hospital where you work :

- **Name of hospital: ___________**
- **City : ___________**
- **Country :**
  - **Netherlands**
  - **Belgium**
- **Type of Hospital:**
  - ❑ Academic
  - ❑ Peripheral

S3/ What kind of (sub)department do you work in?

- ❑ Endocrinology department
- ❑ Nephrology department
- ❑ General internal medicine department
- ❑ Surgery department
- ❑ Other (specify) ___________

S4/ Based on the definition below: How many unique patients suffering from Chronic Hypoparathyroidism have been treated in IN YOUR DEPARTMENT in the year 2016 either managed by yourself or a colleague?

Chronic Hypoparathyroidism

"Hypocalcaemia and inappropriately low parathyroid hormone with a genetic/autoimmune/idiopathic etiology or lasting for more than 12 months after neck surgery, confirmed by repeated laboratory measurements and requiring treatment with vitamin D"

N1 = ____________

If N1 = 0 → OUT

S5/ Based on the definition below: How many patients suffering from Chronic Hypoparathyroidism who are followed IN YOUR DEPARTMENT by yourself or a colleague ARE NOT ADEQUATELY CONTROLLED?

Not adequately controlled chronic hypoparathyroidism

Despite compliance to treatment perceived to be optimal:

Persistence of clinical manifestations/symptoms of hypocalcaemia (amongst others neurological, neuromuscular, cardiovascular, renal)

AND/OR

Biochemical manifestations (one or more of the following criteria):

- *Hypocalcaemia*
- *Significant fluctuations in serum calcium*
- *Hyperphosphatemia*
- *Hypercalciuria*

N2 = …………

If N2 = 0 → Special questionnaire pathway (Section 1 + Section 2 only)

S6/ What is your role in the management of patients with chronic Hypoparathyroidism IN YOUR DEPARTMENT?

- ❑ A. *I treat patients with* chronic Hypoparathyroidism
- ❑ B. I supervise my colleagues / am involved in the treatment decision process.
- ❑ C. *I am not involved in the treatment of* this type of patients

Programming :
If B → 'Please answer the questions in this questionnaire ABOUT HOW YOU WOULD ADVISE your colleagues to treat a chronic hypoparathyroidism patient'
If C → STOP

S7/ Number of physicians who treat chronic hypoparathyroidism working in the same department as you:

N = ___________

S8/ Which (sub)departments in your hospital treat chronic hypoparathyroidism patients?

- ❑ Endocrinology department
- ❑ Nephrology department
- ❑ General internal medicine department
- ❑ Surgery department
- ❑ Other (specify) ____________

PHARMACOVIGILANCE

In accordance with the Pharmaceutical Market Research Code of Conduct and European Medicines Agency good pharmacovigilance practice guidelines, it is our responsibility to report to our client any adverse events, special situations, product quality complaints or reports of drug exposure during pregnancy, on our client's products that become known to us when conducting healthcare market research. Although what is otherwise recorded during this survey will be treated in confidence, should an adverse event (as defined in the Code of Conduct), product quality complaint or reports of drug exposure during pregnancy in a specific patient, or group of patients, be mentioned, we are required to collect this information and report it to the client, even if the event has already been reported to the national health authorities.

To fulfil their responsibilities, for any adverse event / product quality complaint / exposure during pregnancy report recorded, the client has requested completion of the interview, if it is considered appropriate or necessary to do so.

Then we, as the third party conducting this research, would contact you on behalf of the client to collect this information. Of course, you will still have the option to remain anonymous if you so wish.

Are you willing to participate in the interview on this basis?

❑ *Yes I accept* ❑ *No I refuse*

PHYSICIAN PROFILE

Gender: ❑ Male ❑ Female

Age: ______ years

Number of years since specialization as a (specialty in screener):

_________ years

SECTION 1 : GENERAL QUESTIONNAIRE – Specifically related to your chronic hypoparathyroidism patients
(as defined in S4 of the questionnaire)

Q1/ Which of the following biochemical parameters do you use in your screening of chronic hypoparathyroidism?

- ❑ Total calcium
- ❑ Serum ionised calcium
- ❑ Serum albumin
- ❑ Serum phosphate
- ❑ Serum creatinine
- ❑ Serum magnesium
- ❑ PTH
- ❑ 25 (OH) vitamine D
- ❑ 1.25 (OH) vitamine D
- ❑ Urine creatinine
- ❑ Urine calcium
- ❑ Urine magnesium
- ❑ Bone turnover markers
- ❑ Alkaline phosphatase
- ❑ Other, specify…

Q2/ Which additional tests do you use in your diagnosis/screening for complications of chronic hypoparathyroidism?

- ❑ No additional tests
- ❑ Renal ultrasound
- ❑ Renal CT scan
- ❑ 24-hour urinary calcium
- ❑ Total body CT scan
- ❑ Skull X-Ray
- ❑ Abdomen X-Ray
- ❑ Bone densitometry
- ❑ ECG
- ❑ Echocardiography
- ❑ Genetic testing
- ❑ Other, please specify__________

Q3/ **What are the main clinical manifestations and symptoms that you monitor in the follow-up of chronic hypoparathyroidism?**

Neuromuscular :

- ❑ Paraesthesia
- ❑ Cramps
- ❑ Laryngospasm
- ❑ Bronchospasm
- ❑ Severe respiratory disorders
- ❑ Seizures
- ❑ Cerebral calcifications
- ❑ Tetany attacks: ❑ Chvostek's sign ❑ Trousseau's sign (multi-selections)

Cardiovascular :

- ❑ Arrhythmia
- ❑ Palpitations
- ❑ Heart failure

Gastroenterological :

- ❑ Abdominal cramps
- ❑ Constipation

Respiratory :

- ❑ Shortness of breath
- ❑ Wheezing
- ❑ Throat tightness

Neurological :

- ❑ Anxiety
- ❑ Sleep disorder
- ❑ Concentration disorders
- ❑ Mood swings
- ❑ Depression
- ❑ Dementia
- ❑ Fatigue
- ❑ Confusion / cognitive impairment

Renal :

- ❑ Renal failure
- ❑ Polyuria
- ❑ Renal stones
- ❑ Renal calcifications (nephrocalcinosis)

Others:

- ❑ Cataract
- ❑ Papilledema
- ❑ Infection
- ❑ Dental problems
- ❑ Dry mouth or increased thirst
- ❑ Weight loss
- ❑ Fracture
- ❑ Other, please specify: ______________

Q4/ **What do you prescribe as <u>first line pharmacological treatment</u> to patients with chronic hypoparathyroidism?**

- ❑ Calcium supplements
- ❑ Alfacalcidol
- ❑ Calcitriol
- ❑ Dihydrotachysterol
- ❑ Ergocalciferol/cholecalciferol (Vitamin D2/D3)
- ❑ Thiazide diuretic
- ❑ Magnesium salt
- ❑ Phosphate binder
- ❑ No treatment *Programming* ➔ *If ticked, go to Q5 sub 3*
- ❑ Others, specify: ….

PROGRAMMING see Q5 sub 1 or Q5 sub 2

Q5 sub 1/ **<u>Why don't you prescribe Vitamin D</u> as first line treatment to your patients with chronic hypoparathyroidism?**
(question asked only if item 2 AND 3 AND 4 AND 5 NOT ticked in Q4)

____

____

Q5 sub 2/ <u>Why don't you prescribe active vitamin D preparations</u> as first line treatment to your patients with chronic hypoparathyroidism? *(question asked only if items 2 AND 3 AND 4 NOT TICKED in Q4)*

____

____

Q5 sub 3/ **Why don't you prescribe pharmacological treatment as first line treatment?**
(question asked only if "No treatment" ticked in Q4)

____

Q6/ **What other <u>pharmacological treatments</u> would you consider for patients with chronic hypoparathyroidism <u>as second line treatment</u>?**
(Programming: only include options from previous question Q4 that were not ticked)

- ❑ Calcium supplements
- ❑ Alfacalcidol
- ❑ Calcitriol
- ❑ Dihydrotachysterol
- ❑ Ergocalciferol/cholecalciferol (Vitamin D2/D3)
- ❑ Thiazide diuretic
- ❑ Magnesium salt
- ❑ Teriparatide rhPTH(1-34)
- ❑ Phosphate binder
- ❑ Others, specify: ….

Q7/ Do you offer dietary recommendations to your patients with chronic hypoparathyroidism?

- ❑ Yes
  - ❑ Dietary recommendations on salt intake
  - ❑ Dietary recommendations on calcium intake
  - ❑ Dietary recommendations on phosphate intake
  - ❑ Dietary recommendations on magnesium intake
- ❑ No, I do not offer dietary recommendations

Q8/ Could you rank the following treatment goals in the management of chronic hypoparathyroidism from 1 to 8 according to your opinion?
(1 = the most important treatment goal, 8 = the least important treatment goal)

- ❑ Maintenance of serum calcium level, aiming at low normal
- ❑ 24h urinary calcium excretion within reference range
- ❑ Serum phosphate level within reference range
- ❑ Serum calcium-phosphate product below 4.4 $mmol^2/L^2$ (55 mg^2/dl^2)
- ❑ Serum magnesium within reference range
- ❑ Aim at adequate vitamin D status
- ❑ Focus on the overall well-being and QoL of the patient
- ❑ Relieve symptoms of hypocalcaemia

Q9/ How many patients with chronic Hypoparathyroidism as described in this definition do you personally manage:

"Hypocalcaemia and inappropriately low parathyroid hormone with a genetic/autoimmune/idiopathic etiology or lasting for more than 12 months after neck surgery, confirmed by repeated laboratory measurements and requiring treatment with vitamin D"

N = B
Programming : B ≤ N1

Q10/ Could you estimate the subtypes of chronic hypoparathyroidism patients you currently follow (in %) ?

a	❑ Neck surgery	……...%
b	❑ Autoimmune disease	……...%
c	❑ Congenital	…….. %.
d	❑ Irradiation	……...%
e	❑ Idiopathic	……...%
f	❑ Don't know	……...%

TOTAL= 100%

Q11/ **Among the patients suffering from chronic hypoparathyroidism you currently follow, how many are NOT ADEQUATELY CONTROLLED ACCORDING TO YOUR CLINICAL JUDGEMENT despite treatment perceived to be optimal?**

N = C
Programming : C ≤ B

Q12/ **Among the patients suffering from chronic hypoparathyroidism you currently follow, how many are NOT ADEQUATELY CONTROLLED DESPITE TREATMENT PERCEIVED TO BE OPTIMAL BASED ON THE RECOMMENDATIONS from the *"ESE clinical guideline: Treatment of chronic hypoparathyroidism in adults"*** *J Bollerslev, L Rejnmark and others, EJE (2015) 173, G1-G20* **(detailed below):**

- *Serum calcium level (albumin adjusted total calcium or ionized calcium) in the lower part or slightly below the lower limit of the reference range with patients being free of symptoms or signs of hypocalcaemia.*
- *Acceptable symptoms of hypocalcaemia*
- *Adequate vitamin D status.*
- *24-h urinary calcium excretion within the sex-specific reference range.*
- *Serum phosphate levels within the reference range.*
- *Serum calcium–phosphate product below 4.4 mmol²/l² (55 mg²/dl²).*
- *Serum magnesium levels within the reference range.*

N = D
Programming : D ≤ B

Q13/ Among all the patients suffering from chronic hypoparathyroidism you currently follow, how many are NOT ADEQUATELY CONTROLLED ACCORDING TO THE DEFINITION BELOW?

Not adequately controlled chronic hypoparathyroidism

Despite compliance to treatment perceived to be optimal:

Persistence of clinical manifestations/symptoms of hypocalcaemia (amongst others neurological, neuromuscular, cardiovascular, renal)

AND/OR

Biochemical manifestations (one or more of the following criteria):
- *Hypocalcaemia*
- *Significant fluctuations in serum calcium*
- *Hyperphosphatemia*
- *Hypercalciuria*

N = E

Programming : E ≤ B

Programming : if E = 0 → Section 1 + Section 2 only

If E > 0 → Section 1 + Section 2 + Section 3

Q14/ Besides the treatment you normally prescribe to patients with chronic hypoparathyroidism: what pharmacological treatments would you consider for not adequately controlled (as defined in the previous question) patients with chronic hypoparathyroidism ?

- ❑ Calcium supplements
- ❑ Alfacalcidol
- ❑ Calcitriol
- ❑ Dihydrotachysterol
- ❑ Ergocalciferol/cholecalciferol (Vitamin D2/D3)
- ❑ Thiazide diuretic
- ❑ Magnesium salt
- ❑ Teriparatide rhPTH(1-34)
- ❑ Phosphate binder
- ❑ Others, specify: ….

Q15/ Could you select, among the following list, the 3 main causes of difficulties in adequately controlling chronic hypoparathyroidism in your opinion?

- ❑ Belated management of hypoparathyroidism
- ❑ Comorbidities
- ❑ Limited treatment choice
- ❑ Drug interactions
- ❑ Poor compliance
- ❑ Side effects/intolerance of hypoparathyroidism treatments
- ❑ Iatrogenic renal failure
- ❑ Patient on multiple medicines
- ❑ Patients are unable to monitor their serum calcium at home
- ❑ Underdosing of treatments
- ❑ Overdosing of treatments
- ❑ Urolithiasis
- ❑ Other, please specify_______________

Programming : 3 answers max.

Q16/ Could you rank the following risks of not adequately controlled hypoparathyroidism from 1 to 6 according to your opinion?
(1 = the most significant risk, 6 = the least significant risk)

- Renal complications /symptoms /__/
- Neuromuscular complications/symptoms /__/
- Cardiovascular complications/symptoms /__/
- Neurological complications/symptoms /__/
  (For example depression, confusion, weakness, reduced concentration)
- Gastrointestinal complications/symptoms /__/
- Respiratory complications/symptoms /__/

Q17/ **The following figure represents profiles <u>of patients with not adequately controlled chronic hypoparathyroidism</u>.**

B: abnormal biochemical parameters with no/mild clinical symptoms/comorbidities
C: abnormal biochemical parameters with severe clinical symptoms/comorbidities
D: normal biochemical parameters with severe clinical symptoms/comorbidities

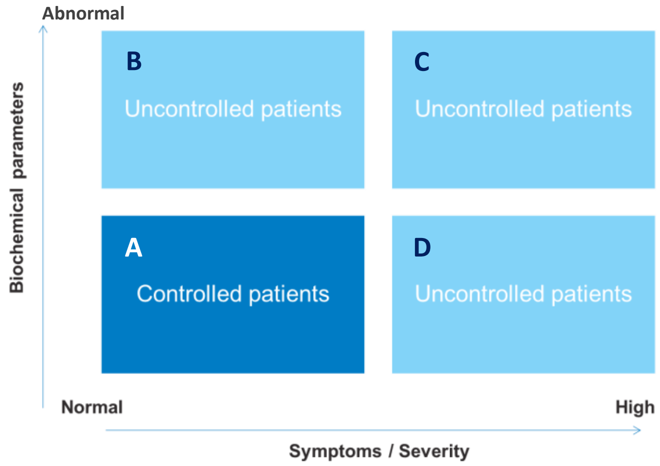

To which extent, in your daily practice, do you consider these three patients' profiles as "Not adequately controlled chronic hypoparathyroidism"?

Q17a/ **Profile B *: "abnormal biochemical parameters with low/normal clinical symptoms/comorbidities"***

☐ *Not at all* ☐ *Somewhat No* ☐ *Somewhat Yes* ☐ *Absolutely*

Q17b/ **Profile C *: "abnormal biochemical parameters with severe clinical symptoms/comorbidities"***

☐ *Not at all* ☐ *Somewhat No* ☐ *Somewhat Yes* ☐ *Absolutely*

Q17c/ **Profile D *: "normal biochemical parameters with severe clinical symptoms/comorbidities"***

☐ *Not at all* ☐ *Somewhat No* ☐ *Somewhat Yes* ☐ *Absolutely*

Q18/ **Could you split your n=B [the number of B patients will be reminded] chronic hypoparathyroidism patients according to the profiles presented in Q17 ? (in %)**

B: abnormal biochemical parameters with no/mild clinical symptoms/comorbidities
C: abnormal biochemical parameters with severe clinical symptoms/comorbidities
D: normal biochemical parameters with severe clinical symptoms/comorbidities

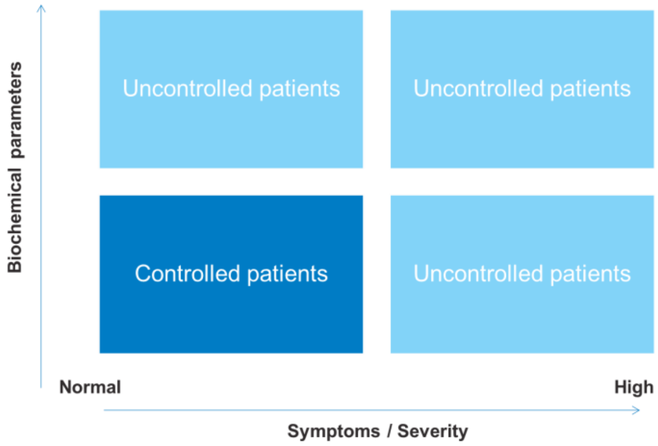

• Patients "controlled"	PROFILE A	.........%
• Patients "not adequately controlled"	PROFILE B	.........%
• Patients "not adequately controlled"	PROFILE C	.........%
• Patients "not adequately controlled"	PROFILE D	.........%

TOTAL = 100%

SECTION 3 : REAL LIFE CLINICAL CASES with not adequately controlled hypoparathyroidism

Only physicians personally involved in the management of patients with not adequately controlled chronic hypoparathyroidism are requested to provide the profile of 2 REAL LIFE CLINICAL CASES of not adequately controlled hypoparathyroidism (as defined below)

Programming : 2 patients among N=E (If E=1 ➔ ask for 1 patient case, if E=0, end of questionnaire)

Since you have declared managing patients with not adequately controlled hypoparathyroidism, we would like you to fill in 1 to 2 real life clinical cases of such patients.This will tremendously help us getting insights into current practices regarding management of not adequately controlled chronic hypoparathyroidism.

Please see below a reminder of the definition of not adequately controlled chronic hypoparathyroidism that we consider in this study

Not adequately controlled chronic hypoparathyroidism

Despite compliance to treatment perceived to be optimal:

Persistence of clinical manifestations/symptoms of hypocalcaemia (amongst others neurological, neuromuscular, cardiovascular, renal)

AND/OR

Biochemical manifestations (one or more of the following criteria):
- *Hypocalcaemia*
- *Significant fluctuations in serum calcium*
- *Hyperphosphatemia*
- *Hypercalciuria*

Not adequately controlled hypoparathyroidism
REAL CLINICAL CASE #1

PATIENT 1 / PROFILE

Q26/ **Age:** |___| years

Q27/ **Gender:** ❑ Female ❑ Male

Q28/ **How long ago was the diagnosis of chronic hypoparathyroidism made?**

|___|___| years AND |___|___| months

For example:

- *2 years and 3 months*
- *0 years and 6 months*
- *3 years and 0 months*

Q29/ **What is the aetiology of the hypoparathyroidism in this patient?** *(you can tick several items)*

❑ Neck surgery complication
⇨ Year of surgery: |___|___|___|___|
❑ Autoimmune disease
❑ Congenital
❑ Irradiation
❑ Idiopathic
❑ Don't know
Programming: more than 1 answer allowed

Q30/ **How often do you see this patient at the out-patient clinic for his/her hypoparathyroidism:**

❑ Every month
❑ Every 2 months
❑ Every 3 months
❑ Twice per year
❑ Once per year
❑ Other, please specify____________

PATIENT 1 / CARE AT TIME OF DIAGNOSIS

Q31/ Were you involved in the diagnosis of chronic hypoparathyroidism or do you have access to the patient's clinical dossier going back to the time of diagnosis?

- ❑ Yes
- ❑ No → PROGRAMMING GO TO Q35

Q32/ The diagnosis of chronic hypoparathyroidism in this patient was established on the basis of:

- ❑ The presence of clinical symptoms which led to a request for a biochemical assessment
- ❑ Random discovery in a biochemical assessment
- ❑ Systematic biochemical assessment carried out after neck surgery
- ❑ Other, please specify____

Programming: 1 answer only

Q32 sub 1/ *Only ask if item 1 in Q32 (presence of clinical symptoms)*

What were the clinical manifestations and symptoms that raised suspicion about the diagnosis? *(Programming: multiple choice)*

Neuromuscular:

- ❑ Paraesthesia
- ❑ Cramps
- ❑ Bronchospasm
- ❑ Seizures
- ❑ Cerebral calcifications
- ❑ Tetany attacks: ❑ Chvostek's sign
- ❑ Laryngospasm
- ❑ Severe respiratory disorders
- ❑ Trousseau's sign (programming multi-selections)

Cardiovascular:

- ❑ Arrhythmia
- ❑ Heart failure
- ❑ Palpitations

Gastroenterological:

- ❑ Abdominal cramps
- ❑ Constipation

Respiratory:

- ❑ Shortness of breath
- ❑ Wheezing
- ❑ Throat tightness

Neurological:

- ❑ Anxiety
- ❑ Concentration disorders
- ❑ Depression
- ❑ Dementia
- ❑ Fatigue
- ❑ Confusion / cognitive impairment
- ❑ Sleep disorder
- ❑ Mood swings

Renal:

- ❑ Renal failure
- ❑ Polyuria
- ❑ Renal stones
- ❑ Renal calcifications (nephrocalcinosis)

Others:

- ❑ Cataract
- ❑ Papilledema
- ❑ Infection
- ❑ Dental problems
- ❑ Dry mouth or increased thirst
- ❑ Weight loss
- ❑ Fracture
- ❑ Other, please specify: ______________

Q33/ **Which comorbidities did this patient have at the time of diagnosis:**

- ❑ No comorbidity
- ❑ Renal comorbidity:
  - ❑ Renal calculi
  - ❑ Nephrocalcinosis
  - ❑ CKD Stage 1 (structural anomalies)
  - ❑ CKD Stage 2 (mild RF)
  - ❑ CKD Stage 3 (moderate RF)
  - ❑ CKD Stage 4 (severe RF)
  - ❑ CKD Stage 5 (end-stage RF)
- ❑ Hypertension
- ❑ Cancer
- ❑ Heart failure
- ❑ Dyslipemia
- ❑ DiGeorge syndrome
- ❑ Calciphylaxis
- ❑ Excess weight
- ❑ Diabetes
- ❑ Other, please specify_____________

Q34/ What were the treatments prescribed after the diagnosis of chronic hypoparathyroidism was established:

- ❑ Calcium supplements: _______ mg/day
- ❑ Alfacalcidol: _____ µg/day
- ❑ Calcitriol: _______ µg/day
- ❑ Dihydrotachysterol
- ❑ Ergocalciferol/cholecalciferol (Vitamin D2/D3)
- ❑ Thiazide diuretic
- ❑ Magnesium salt
- ❑ Phosphate binder
- ❑ Teriparatide rhPTH(1-34)
- ❑ No treatment Programming ➔ If ticked, go to Q34 sub 3
- ❑ Others, specify: ….

PROGRAMMING see Q34 sub 1 or Q34 sub 2

Programming: several answers can be given, unless "No treatment" is selected
Number of decimals allowed:

- *Calcium salts, magnesium salts: 0 decimal*
- *Alfacalcidol, Calcitriol: 2 decimals*

	Min limit	Max limit
Calcium salts	-	15000
Alfacalcidol	-	10
Calcitriol	-	10

Q34 sub 1/ **Why didn't you prescribe vitamin D to this patient?**
(question asked ONLY IF ITEM 2 AND 3 AND 4 AND 5 NOT TICKED in Q34)

___

___

___

Q34 sub 2/ **Why didn't you prescribe active vitamin D preparations to this patient?**
(question asked ONLY IF ITEMS 2 AND 3 AND 4 NOT TICKED in Q34)

___

___

___

Q34 sub 3/ **Why didn't you prescribe any pharmacological treatment?**
(question asked only if "No treatment" ticked in Q34)

___

___

___

PATIENT 1 / CURRENT CARE

Q35/ What clinical manifestations (symptoms/signs) of chronic hypoparathyroidism does this patient currently have?

Neuromuscular:
- ❑ Paraesthesia
- ❑ Cramps
- ❑ Laryngospasm
- ❑ Bronchospasm
- ❑ Severe respiratory disorders
- ❑ Seizures
- ❑ Cerebral calcifications
- ❑ Tetany attacks: ❑ Chvostek's sign ❑ Trousseau's sign (multi-selections)

Cardiovascular:
- ❑ Arrhythmia
- ❑ Palpitations
- ❑ Heart failure

Gastroenterological:
- ❑ Abdominal cramps
- ❑ Constipation

Respiratory:
- ❑ Shortness of breath
- ❑ Wheezing
- ❑ Throat tightness

Neurological:
- ❑ Anxiety
- ❑ Sleep disorder
- ❑ Concentration disorders
- ❑ Mood swings
- ❑ Depression
- ❑ Dementia
- ❑ Fatigue
- ❑ Confusion / cognitive impairment

Renal :
- ❑ Renal failure
- ❑ Polyuria
- ❑ Renal stones
- ❑ Renal calcifications (nephrocalcinosis)

Others:
- ❑ Cataract
- ❑ Papilledema
- ❑ Infection
- ❑ Dental problems
- ❑ Dry mouth or increased thirst
- ❑ Weight loss
- ❑ Fracture
- ❑ Other, please specify: ______________

Q36/ **Does this patient currently still have biochemical parameters outside the normal laboratory reference ranges?**

- ❑ YES
- ❑ NO

Q36 sub 1/ **If Yes, which parameters are currently outside the normal laboratory reference ranges?**

- ❑ Total calcium
- ❑ Serum ionised calcium
- ❑ Serum albumin
- ❑ Serum phosphate
- ❑ Serum creatinine
- ❑ Serum magnesium
- ❑ 25 (OH) vitamine D
- ❑ 1.25 (OH) vitamine D
- ❑ Urine creatinine
- ❑ Urine calcium
- ❑ Urine magnesium
- ❑ Bone turnover markers
- ❑ Alkaline phosphatase
- ❑ Other, specify…

Q37/ **How often do you monitor biochemical parameters in this patient?**

- ❑ Every month
- ❑ Every 2 months
- ❑ Every 3 months
- ❑ Twice per year
- ❑ Once per year
- ❑ Other, please specify___________

Q38/ What are the patient's current comorbidities?

- ❑ No comorbidity
- ❑ Renal comorbidity:
  - ❑ Renal calculi
  - ❑ Nephrocalcinosis
  - ❑ Nephrolithiasis
  - ❑ CKD Stage 1 (structural anomalies)
  - ❑ CKD Stage 2 (mild RF)
  - ❑ CKD Stage 3 (moderate RF)
  - ❑ CKD Stage 4 (severe RF)
  - ❑ CKD Stage 5 (end-stage RF)
- ❑ Hypertension
- ❑ Cancer
- ❑ Heart failure
- ❑ Dyslipidemia
- ❑ DiGeorge syndrome
- ❑ Calciphylaxis
- ❑ Excess weight
- ❑ Diabetes
- ❑ Other, please specify_____________

Q39/ Which tests do you currently carry out to monitor the hypoparathyroidism?

- ❑ Renal ultrasound
- ❑ Renal CT scan
- ❑ 24-hour urinary calcium
- ❑ Total body CT scan
- ❑ Skull X-Ray
- ❑ Abdomen X-Ray
- ❑ Bone densitometry
- ❑ ECG
- ❑ Echocardiography
- ❑ Genetic testing
- ❑ None
- ❑ Other, please specify__________

Q40/ How often is this test carried out: *(Programming: for each test selected in Q39)*

- ❑ Every month
- ❑ Every 2 months
- ❑ Every 3 months
- ❑ Twice per year
- ❑ Once per year
- ❑ Other, please specify____________

Q41/ What treatment is this patient currently taking to control his/her hypoparathyroidism?

- ❑ Calcium supplements: ______ mg/day
- ❑ Alfacalcidol ____ µg/day
- ❑ Calcitriol: _______ µg/day
- ❑ Dihydrotachysterol
- ❑ Ergocalciferol/cholecalciferol (Vitamin D2/D3)
- ❑ Thiazide diuretic
- ❑ Magnesium salt
- ❑ Phosphate binder
- ❑ Teriparatide rhPTH(1-34)
- ❑ No treatment
- ❑ Others, specify: ….

Programming: several answers can be given, unless "No treatment" is selected
Number of decimals allowed:

- *Calcium supplements, magnesium salts: 0 decimal*
- *Alfacalcidol, Calcitriol: 2 decimals*

	Min limit	Max limit
Calcium supplements	-	15000
Alfacalcidol	-	10
Calcitriol	-	10

Q42/ **Have you had to increase the medication dosages to try to control your patient's hypoparathyroidism?**

- ❑ Yes, I increased the calcium dosage and the patient is still not adequately controlled
- ❑ Yes, I increased the dosage of vitamine D and the patient is still not adequately controlled
- ❑ Yes, I increased both the calcium and the vitamine D dosage and the patient is still not adequately controlled
- ❑ No, I did not increase the dosage for the following reasons
  - ☐ Maximum daily dose achieved for calcium, at the daily dose of _____
  - ☐ Renal clearance was too low to increase the doses
  - ☐ Other, please specify__________

  Programming: Multiple answers possible

Q43/ Did this patient need to be hospitalized over the past 12 months because of inability to control chronic hypoparathyroidism?

❑ Yes ❑ No

Q43 sub 1/ *If yes, in Q43*

How many times was the patient hospitalized in the past 12 months?

|___| times

Q43 sub 2/ *If yes, in Q43*

How long in total was the patient hospitalized for in the past 12 months?

|___|___| Days OR |___|___| Week(s) OR |___|___| Month(s)

Q44/ **Can you rank the degree of change in this patient's quality of life due to hypoparathyroidism since the onset of the disease,?**

(= : no change at all / ⬂ = diminution of quality of life, ⬀ = improvement of quality of life)

⬂ = ⬀

Q45/ **In your opinion, what is the patient's level of compliance with his/her prescribed treatment for hypoparathyroidism?** (on a scale of 0 to 10)

(0 = not compliant at all, 10 = very compliant)

0 5 10

Q45bis/ *If results ≤ 5 in Q45*

In your opinion, what is the main reason for this patient's non-compliance to treatment?

❑ Intolerance/side effects of the medication
❑ Tiredness of the treatment due to the number of sachets/tablets/capsules to be taken every day
❑ Absence of symptoms
❑ Disease not really taken seriously
❑ Others, please specify: ____________________
❑ Don't know

Programming : single choice question

Q46/ To summarize, in this patient, today:

- **CLINICAL SYMPTOMS related to hypoparathyroidism are**
  ❑ No symptoms ❑ Not important ❑ Important ❑ Very important
- **Diagnostic BIOCHEMICAL PARAMETERS for hypoparathyroidism are**
  ❑ Normal ❑ Slightly disturbed ❑ Disturbed ❑ Strongly disturbed
- **COMORBIDITIES are**
  ❑ No comorbidities ❑ Not important ❑ Important ❑ Very important
- **The burden caused by use of prescribed MEDICATION is**
  ❑ No medication prescribed ❑ Not important ❑ Important ❑ Very important

Q47/ For how long has this patient's hypoparathyroidism been not adequately controlled despite optimal treatment?

Number of years = |___|___| AND number of months = |___|___|

For example:

- *2 years and 3 months*
- *0 years and 6 months*
- *3 years and 0 months*

Q48/ In your opinion, what are the main causes for not being able to adequately control chronic hypoparathyroidism in this patient?

❑ Belated management of hypoparathyroidism
❑ Comorbidities
❑ Limited treatment choice
❑ Drug interaction
❑ Poor compliance
❑ Side effects/intolerance of hypoparathyroidism treatments
❑ Iatrogenic renal failure due to calcium overdose
❑ Patient on multiple medicines
❑ Underdosing of treatments
❑ Urolithiasis
❑ Other, please specify_______________
❑ Don't know/No explanation

Programming: several answers can be given, unless "Don't know/No explanation" selected

Q49/ **In your opinion, which profile of not adequately controlled chronic hypoparathyroidism illustrated in the following figure corresponds best with that of your patient?**

Profile B: abnormal biochemical parameters with low/normal clinical symptoms/comorbidities
Profile C: abnormal biochemical parameters with severe clinical symptoms/comorbidities
Profile D: normal biochemical parameters with severe clinical symptoms/comorbidities

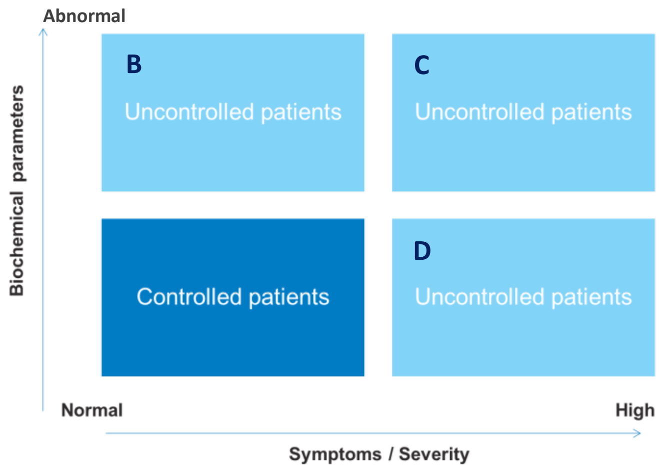

- ❑ **Profile B**
- ❑ **Profile C**
- ❑ **Profile D**

(1 answer possible)

Not adequately controlled hypoparathyroidism
REAL CLINICAL CASE #2

SAME QUESTIONS AS FOR PATIENT CASE 1
